# Should We Fear Wipe-Out in Glaucoma Surgery?

**DOI:** 10.3390/diagnostics15131571

**Published:** 2025-06-20

**Authors:** Marco Zeppieri, Ludovica Cannizzaro, Giuseppe Gagliano, Francesco Cappellani, Lorenzo Rapisarda, Alfonso Spinello, Antonio Longo, Andrea Russo, Alessandro Avitabile

**Affiliations:** 1 Department of Ophthalmology, University Hospital of Udine, 33100 Udine, Italy; 2 Department of Medicine, Surgery and Health Sciences, University of Trieste, 34127 Trieste, Italy; 3 Department of Ophthalmology, University of Catania, 95123 Catania, Italy; 4 Department of Medicine and Surgery, University of Enna “Kore”, Piazza dell’Università, 94100 Enna, Italy; 5 Mediterranean Foundation “G.B. Morgagni”, Via Sant’Euplio, 95100 Catania, Italy; 6 ASP 8 Siracusa, PO Umberto I, 96100 Siracusa, Italy

**Keywords:** wipe-out, glaucoma, MIGS, vision loss

## Abstract

Wipe-out is defined as a sudden, unexplained, and irreversible loss of residual central vision following glaucoma surgery, typically in eyes with advanced visual field damage and severely compromised optic nerves. The purpose of this review is to critically assess the current incidence, risk factors, pathophysiological mechanisms, and clinical relevance of “wipe-out”, a rare but devastating complication of glaucoma surgery characterized by sudden, unexplained central vision loss postoperatively. A comprehensive literature review was conducted, analyzing key peer-reviewed studies from electronic databases (PubMed, Medline, and Google Scholar) published up to 2025. The data from the literature published prior to the year 2000 suggest that wipe-out incidences range broadly from <1% to 13%. Contemporary prospective studies and large-scale reviews indicate a significantly lower current incidence, frequently below 1%. Identified risk factors include severe preoperative visual field loss (especially split fixation), older age, immediate postoperative hypotony, and compromised optic nerve head perfusion. The proposed mechanisms involve acute vascular insults, ischemia–reperfusion injury, and accelerated apoptosis of already vulnerable retinal ganglion cells. Modern MIGS and refined trabeculectomy techniques exhibit notably lower wipe-out risks compared to historical data. The literature emphasizes preventive management, including careful patient selection, incremental intraocular pressure reduction, and minimally invasive anesthetic approaches. Although wipe-out syndrome represents a serious complication, its incidence in modern glaucoma surgery is minimal. The considerable benefits of contemporary surgical approaches—particularly MIGS—in preserving vision clearly outweigh this very low risk. Ophthalmologists should remain vigilant but confident in the safety and efficacy of modern glaucoma surgical techniques, emphasizing proactive intervention to prevent blindness rather than avoiding necessary surgery in consideration of the minimal risk of wipe-out.

## 1. Introduction

Glaucoma is a chronic, progressive optic neuropathy characterized by irreversible retinal ganglion cell loss; it is widely regarded as a multifactorial “complex trait” disease. Its development reflects the interplay of numerous genetic polymorphisms and non-genetic influences (e.g., aging, vascular dysregulation, and oxidative stress) since known gene variants explain only a fraction of cases [1,2]. Emerging evidence indicates that epigenetic mechanisms—including DNA methylation, histone modification, and noncoding RNAs—are key mediators linking genetic susceptibility with environmental triggers in glaucoma pathogenesis [1,3,4]. Accordingly, when the intraocular pressure cannot be adequately controlled by maximal medical or laser therapy—particularly in progressive or advanced disease—surgical intervention (e.g., trabeculectomy or glaucoma drainage surgery) is generally required to preserve vision [5,6,7].

“Wipe-out” (also known as the “snuff-out” phenomenon) refers to an idiopathic, irreversible loss of central vision that occurs in the immediate postoperative period after glaucoma surgery, typically in eyes with advanced glaucoma [6,8,9]. In practical terms, a patient with severe glaucoma who still has a residual central island of vision may lose that remaining vision suddenly after an otherwise successful surgery, with no other identifiable cause such as overt surgical complications or ocular pathology [9]. This rare complication is devastating for both patients and surgeons as it essentially represents a total wipe-out of the patient’s residual vision [9,10].

Historically, wipe-out was a feared outcome in the surgical management of end-stage glaucoma. Early reports and anecdotes in the 1970s–1980s suggested that performing a trabeculectomy (the standard filtering surgery) in an eye with only a tiny central visual field could result in blindness, with quoted incidences as high as 13% in some series [11,12]. For example, in 1986, Aggarwal and Hendeles first described the “risk of sudden visual loss” after trabeculectomy in advanced primary open-angle glaucoma, raising an alarm among ophthalmologists [12]. Such concerns led many surgeons to hesitate before operating on the last seeing eye of glaucoma patients with advanced visual field loss [10]. In clinical lore, it was often said that a patient with only a central island of vision might be better off without surgery (to avoid wipe-out), even if the intraocular pressure (IOP) was high and not manageable with pharmacological approaches [10]. This conservative attitude persisted for years.

In recent decades, glaucoma surgery has seen significant evolution, particularly with the introduction of minimally invasive glaucoma operations (MIGSs) and minimally invasive bleb-forming surgeries (MIBSs). MIGSs include angle-based techniques (e.g., iStent, Hydrus Microstent, and trabecular bypass), whereas MIBSs encompass subconjunctival filtering devices like the XEN Gel Stent and PreserFlo MicroShunt, which establish an external filtration bleb [13,14]. MIGSs are a group of newer, micro-incisional procedures designed to lower the IOP with less tissue trauma and risk than traditional trabeculectomy or tube shunt surgeries [13,14]. Over the past ten to fifteen years, an unprecedented growth in MIGS options has “bridged the gap” between medical or laser therapy and more invasive filtering surgeries [14,15]. MIGS procedures (such as the iStent, Hydrus Microstent, trabecular bypass or goniotomy procedures, XEN gel stent, PreserFlo MicroShunt, etc.) aim to achieve significant IOP reduction with a far safer profile and faster recovery [16,17]. They typically avoid the creation of an external bleb or full-thickness scleral fistula, thereby minimizing hypotony (excessively low IOP) in the early postoperative period—a factor thought to be implicated in wipe-out [18,19]. MIGSs have transformed the surgical approach to glaucoma by enabling intervention earlier in the disease course with less risk [13,14]. In fact, one large meta-analysis of MIGS outcomes reported that no cases of severe vision loss attributable to glaucoma surgery occurred in over 2900 eyes treated with various MIGS procedures, with the most frequent complications being mild issues like IOP spikes [13,14,20].

Given these advances, there is renewed interest in whether wipe-out remains a relevant clinical concern today. MIGSs now allow us to operate on glaucoma patients at moderate stages, potentially delaying or avoiding the need for high-risk end-stage surgeries [13,15]. Even when traditional surgery is needed for advanced glaucoma, modern surgical techniques and a better understanding of risk factors may have reduced the incidence of wipe-out to a very low level [5,6,21,22]. Some have even questioned if the wipe-out phenomenon was overstated or is now “just a blast from the past” [8]. Nonetheless, be-cause wipe-out—however rare—results in permanent blindness, it continues to inspire caution [9,23]. This review will delve into the current evidence on wipe-out in glaucoma surgery, especially in the context of MIGS, to answer the following question: should we still fear wipe-out? We will define the phenomenon and its historical context, examine the latest data on incidence and risk factors, and discuss how MIGS and other innovations impact our perspective on this complication.

## 2. Materials and Methods

A comprehensive review of the literature was conducted to identify and analyze relevant studies concerning the “wipe-out” phenomenon associated with glaucoma surgery. The systematic search was carried out using electronic databases, including PubMed, Medline, and Google Scholar. Keywords employed during the search included “wipe-out glaucoma”, “snuff-out glaucoma”, “visual loss after trabeculectomy”, “vision loss glaucoma surgery”, “advanced glaucoma surgical outcomes”, and “minimally invasive glaucoma surgery (MIGS) safety”.

The search included peer-reviewed publications in the English language, spanning from the initial descriptions of the wipe-out phenomenon in the 1970s to contemporary studies and reviews published up to 2025. Special emphasis was placed on studies published from 2010 onwards to reflect the advent and widespread adoption of minimally invasive glaucoma surgeries (MIGSs). Both retrospective and prospective clinical studies, systematic reviews, meta-analyses, case reports, expert opinion pieces, consensus statements, and official guidelines were reviewed to provide a comprehensive assessment of wipe-out incidence, potential mechanisms, risk factors, and management strategies.

Inclusion criteria for the selected literature were as follows: (1) articles specifically addressing wipe-out or unexplained severe vision loss occurring shortly after glaucoma surgery; (2) studies evaluating the safety profiles and incidence of visual complications in traditional glaucoma surgeries or MIGS procedures or studies including comparative analyses of both; and (3) publications discussing pathophysiological hypotheses, preventive strategies, or management of vision loss in advanced glaucoma surgical cases. Studies primarily focusing on identifiable surgical complications (e.g., hemorrhage, infection, or known structural complications) resulting in vision loss were excluded as the scope of this review was restricted to idiopathic wipe-out.

Articles were initially screened based on titles and abstracts. Relevant studies underwent detailed full-text evaluation, and additional pertinent references cited within these studies were also reviewed for inclusion. The selected literature was critically analyzed and synthesized, with findings grouped thematically to clearly present evidence regarding the epidemiology, clinical characteristics, proposed etiological mechanisms, and preventive approaches of the wipe-out phenomenon. This methodological approach ensured a robust and comprehensive foundation for the subsequent analysis and discussion within this review.

## 3. Results

### 3.1. Incidence of Wipe-Out in Glaucoma Surgery

Early reports and historical data: Wipe-out has always been considered uncommon, but past studies reported a wide range of incidence figures. Early retrospective analyses suggested that a small percentage of advanced glaucoma eyes might suffer unexplained vision loss after filtration surgery [8,9,11]. Costa et al. (1993) provided one of the first detailed examinations of this phenomenon in a cohort of patients who underwent trabeculectomy [10]. In their series of 508 operated eyes, they identified four eyes (0.8%) in which central visual acuity loss occurred without an obvious cause—consistent with wipe-out. By contrast, the majority of vision loss cases in that study were due to explainable causes such as cataract formation (16 eyes) or hypotony maculopathy (6 eyes). The Costa study is often cited because it confirmed that wipe-out “does exist” but is rare, and it established a baseline incidence of under 1% in a large sample [10]. Around the same period, anecdotal reports had suggested higher rates in certain high-risk groups. For example, Kolker (1977) in a much earlier analysis had warned that up to 13.6% of end-stage glaucoma eyes could lose vision post-surgery, though such high rates were likely overestimates from small samples or confounded cases [11]. Similarly, Aggarwal and Hendeles (1986) described several instances of sudden blindness after trabeculectomy in advanced glaucoma, fueling the notion that wipe-out, while not inevitable, is a tangible risk when operating on eyes with only a residual central field [12]. By the early 2000s, estimates of wipe-out frequency ranged roughly from <1% to about 5% in advanced cases, with occasional outliers reporting higher figures [8,9,12]. This uncertainty perpetuated a cautious approach. However, more recent evidence suggests that the frequency of true wipe-out is much lower than once feared [5,18].

Prospective studies in advanced glaucoma: The first prospective investigation dedicated to this topic was conducted by Topouzis et al. (2005) [9]. They enrolled 21 patients with end-stage glaucoma (mean visual field mean deviation ~ –28 dB, i.e., very advanced) who underwent trabeculectomy with mitomycin-C and carefully monitored their central vision and fields for wipe-out. Crucially, not a single case of wipe-out was observed—after 3 months post-op, no patient had an irreversible loss of central vision; on average, visual acuity and central visual field sensitivity were unchanged from pre-op [9]. The IOP was effectively lowered (~14 mmHg drop) without significant intraoperative issues (some transient hypotony occurred but was managed). The authors concluded that in this consecutive series of end-stage cases, surgery “preserved vision with no occurrences of ‘wipe-out’” [9]. This was a landmark finding: it suggests that with modern microsurgical techniques and careful case selection, the risk of wipe-out could be essentially zero, even in glaucoma eyes with the worst conditions.

An accompanying editorial by Moster echoed this, pointedly asking whether wipe-out might be more of a historical concern rather than a uncommon contemporary complication [8]. Further prospective evidence comes from Bhadra et al. (2022), who specifically evaluated advanced glaucoma eyes with split fixation (meaning the central 5–10° of the visual field was threatened) undergoing filtration surgery [24]. In their study of 30 such eyes, they similarly found no instances of wipe-out after surgery [24]. A couple of eyes did lose two lines of vision transiently due to cataract progression or choroidal detachment, but these had identifiable causes and were not permanent. The conclusion was that wipe-out is a truly rare phenomenon—even among high-risk eyes, none had an unexplained permanent central vision loss [24]. These prospective findings, along with other series (e.g., Leleu et al. 2019 examining deep sclerectomy in severe glaucoma, which also reported zero cases of wipe-out), reinforce that the incidence of wipe-out in the modern era is on the order of 0–1% or “very low” [19].

Wipe-out with modern MIGS and tube shunts: What about newer surgeries? MIGS procedures were designed to be safer, so one would expect an extremely low (possibly zero) incidence of wipe-out with MIGS [13,15]. Indeed, in the collective MIGS literature up to 2020, there are no reports of classic wipe-out [13,15]. Large reviews and trials of MIGS (mostly performed in eyes with mild to moderate glaucoma in combination with cataract surgery) have not documented any cases of unexplained permanent central vision loss attributable to surgery [13,15]. For instance, a 2017 systematic review (Lavia et al.) covering thousands of MIGS cases noted that no loss of best-corrected acuity due to glaucoma surgery occurred; most complications were minor, such as transient IOP spikes, with zero cases of “snuff-out” [13,14]. This is perhaps not surprising, as MIGSs typically produce a more modest IOP reduction and avoid extreme hypotony [13,14]. However, MIGSs are also usually performed on less advanced patients, so the situation of an end-stage eye undergoing MIGS has been relatively uncommon until recently [16,17].

It is only now, as surgeons gain confidence with MIGSs, that some MIGSs are being tried in more advanced glaucoma cases—and in such scenarios, rare complications like wipe-out can still occur. Notably, Ozmen et al. (2025) reported what is believed to be the first case of wipe-out following an MIGS procedure [25]. In their case report, a 62-year-old man with pseudoexfoliation glaucoma and very advanced damage underwent an uncomplicated gonioscopy-assisted transluminal trabeculotomy (GATT, an ab-interno canalotomy MIGS) combined with cataract surgery. In the immediate postoperative period, he experienced a significant decrease in central visual acuity and visual field, consistent with a wipe-out phenomenon. All other potential causes were ruled out, and the authors concluded that this was indeed wipe-out triggered by the GATT procedure—the first such instance documented for GATT + phaco [25]. Similarly, in a series of 44 advanced glaucoma eyes treated with GATT, only one patient (2.3%) suffered a severe vision loss complication, which was a wipe-out in an individual with degenerative high myopia [25]. While these are isolated occurrences, they remind us that no surgery is completely without risk. Even MIGS, if performed in an eye with almost no visual reserve, can on rare occasion precipitate wipe-out.

Meanwhile, outcomes from glaucoma drainage implants (tube shunts) have not prominently featured wipe-out as a complication in published studies [26]. Tubes can cause early hypotony (especially non-valved implants), but many surgeons tie off non-valved tubes to prevent immediate flow. It stands to reason that the incidence of wipe-out with modern tube surgery is comparably low, though the literature specifically addressing wipe-out with tubes is scant [7,26,27]. In the Treatment of Advanced Glaucoma Study (TAGS)—a multicenter trial comparing primary tube shunt vs. trabeculectomy in advanced glaucoma—there was no significant occurrence of wipe-out in either arm; at 5 years, surgical patients had better IOP control and similar visual outcomes as those managed medically [21]. In fact, by 5 years, the TAGS data showed that primary surgery (trabeculectomy) prevented disease progression more effectively than medication in advanced cases without causing excess vision loss on average [21].

In summary, across all surgical modalities, the incidence of true wipe-out is very low—on the order of well under 1% in most contemporary reports. Older studies in the literature reporting higher percentages likely included other causes of vision loss or were limited by small sample sizes [11,12,24]. The best evidence today indicates that wipe-out is a real but exceptionally rare complication of glaucoma surgery.

### 3.2. Risk Factors Associated with Wipe-Out

Although wipe-out can sometimes seem capricious, several risk factors have been identified that predispose an eye to this complication. The unifying theme is an eye with severely compromised functional reserve—essentially, an optic nerve and retina already on the brink of complete failure [10,22,24].

- Advanced visual field loss (split fixation): By definition, wipe-out has been observed almost exclusively in advanced or end-stage glaucoma eyes [24]. A classic risk factor is a preoperative visual field showing central vision involvement, such as a split fixation or only a small central island remaining [10]. Costa et al. found that eyes with preoperative “macular splitting” on the visual field (i.e., the horizontal meridian through the central 5° had a defect in one hemifield) were significantly more likely to experience wipe-out [10]. Essentially, if the glaucoma has encroached on the central 10° of the field, the optic nerve’s reserve is minimal, and any further insult may abolish the remaining vision. A recent study in Japan also identified specific central visual subfield sensitivity as being predictive of future central vision loss in advanced glaucoma, reinforcing that the involvement of the central field is a red flag (though that study was about progression in general, and not just post-op occurrences) [28]. In practical terms, surgeons recognize that an eye with only a “tubular” field or a lone temporal island is at potential risk—these are exactly the scenarios in which wipe-out has been reported [29]. Patients with split fixation were the focus of Bhadra et al.’s 2022 prospective study; while none of their patients ended up with wipe-out, the fact that such a study was deemed important shows that this subgroup is considered high-risk [24]. Case reports of wipe-out (including MIGS cases) invariably involve eyes that had severe glaucomatous damage pre-surgery [25].
- Older age: Age-related factors appear to play a role. Costa et al. noted that older patients were more likely to suffer wipe-out, with the average age of wipe-out cases being higher than that for cases without [10]. Aging may contribute to less resilient optic nerve perfusion or greater susceptibility to IOP changes and ischemia [8,30]. An older optic nerve head might have impaired autoregulatory capacity, for instance [2]. However, age is likely a proxy for other factors (long-standing disease, vascular health, etc.) rather than a direct cause.
- Early postoperative hypotony: Several findings point to a low intraocular pressure in the immediate postoperative period as a risk factor. In Costa’s study, eyes that had severe hypotony on day 1 (IOP ≤2 mmHg) were significantly more likely to experience wipe-out [10]. Hypotony—an extremely low IOP—can compromise ocular perfusion pressure or cause structural changes (e.g., choroidal effusions and retinal folds) that might damage central vision [29]. In Topouzis et al.’s series, they carefully avoided sustained hypotony, which may partly explain the absence of wipe-out [9]. The association between immediate hypotony and wipe-out risk suggests that a sudden, drastic drop in the IOP could precipitate the phenomenon in a susceptible eye [10,29]. This has parallels to decompression retinopathy, a condition where a sharp IOP reduction causes retinal hemorrhages and can affect vision [18,31]. Indeed, case reports exist of decompression retinopathy after glaucoma surgery causing central vision loss (Karadimas 2002), which may overlap with what we call wipe-out [18]. Hypotony might lead to temporary retinal/choroidal changes which, in an end-stage eye, become permanent vision loss [10]
- Perioperative ocular ischemia: Wipe-out might sometimes result from a vascular insult to the optic nerve or retina during surgery [1]. One often-cited risk factor is the use of retrobulbar anesthesia, which in rare cases can cause optic nerve or central retinal artery compromise (for example, via injection pressure or vasospasm) [31]. There is a documented instance of a patient who had a central retinal artery occlusion presumably from a retrobulbar block during glaucoma surgery, leading to irreversible vision loss [31]. While modern anesthesia techniques (sub-Tenon’s or topical anesthesia) have largely mitigated this, it remains a consideration [18]. Patients with known vascular risk factors (e.g., severe hypertension, diabetes, and carotid artery disease) might have less capacity to tolerate fluctuations in ocular perfusion [1,2]. Some authors have postulated that an acute ischemic optic neuropathy (an infarct in the remaining neuroretinal rim) could underlie wipe-out in certain cases, analogous to how some cardiac surgeries can cause anterior ischemic optic neuropathy in susceptible patients [18,30]. However, concrete evidence linking systemic vascular health to wipe-out is limited. One retrospective finding was that patients with systemic cardiovascular disease had a higher incidence of hypotony maculopathy (not wipe-out per se), which suggests that vascular issues could exacerbate hypotony effects [2].
- Optic nerve vulnerability: Not surprisingly, an optic nerve that is already severely damaged (thin neuroretinal rim and pale appearance) is at risk [4]. Some glaucoma specialists anecdotally mention that an extremely “fragile” optic disk (for example, very advanced cupping with barely any rim) raises concern. While difficult to quantify, this speaks to the intrinsic robustness of the remaining ganglion cells [22]. It is possible that certain optic nerves—due to genetic or structural factors—are less tolerant of surgical perturbations [4]. For instance, eyes with high myopia have stretched optic nerves and poorer blood flow; the wipe-out case in a degenerative myope from the GATT series supports that high myopia might amplify risk [25].
- Type of surgery and surgical technique: Traditional full-thickness filtering surgeries (like older trephinations or uncontrolled full-thickness procedures) carry a higher hypotony risk than modern guarded trabeculectomy, and indeed, wipe-out was mostly discussed in the era of full-thickness procedures [11,12]. Modern trabeculectomy with adjustable sutures and anti-fibrotics allows for better IOP titration [6,32]. Non-penetrating surgeries (deep sclerectomy and canaloplasty) inherently avoid sudden decompression, which likely lowers wipe-out risk—Leleu et al. (2019) found no wipe-out cases after deep sclerectomy in a series of severe glaucoma patients [19]. MIGS procedures vary: ab-interno trabecular bypass (iStent, Trabectome) produce gradual IOP reduction and have essentially no wipe-out reports, whereas procedures that can cause larger immediate outflow (GATT and XEN stent) theoretically have a bit more risk (and indeed, the rare MIGS wipe-outs were observed with GATT and a bleb shunt) [17,20,25,33]. Combined surgeries (e.g., phacoemulsification + glaucoma surgery) could have an interplay: cataract surgery can induce inflammation or IOP changes, but generally, phaco tends to improve perfusion by gradually lowering the IOP [16,17]. A British survey (Ramsden et al. 2023) even looked at wipe-out after cataract surgery alone in glaucoma patients and found it exceedingly rare, reinforcing that the surgical IOP drop is a key factor [23].

To summarize risk factors, an advanced glaucomatous eye in an older patient with central field loss and a very tenuous optic nerve, experiencing a drastic postoperative IOP drop or an ocular vascular event, is the quintessential scenario for wipe-out [10,24,25]. Fortunately, we can often identify such eyes preoperatively. In practice, surgeons stratify risk by visual field status—eyes with only a small central field are flagged. It is in these eyes that extra caution and preventive measures (discussed below) are concentrated. The risk factors are listed in Table 1.

### 3.3. Pathophysiological Mechanisms

The exact pathophysiology of wipe-out remains somewhat enigmatic as by definition, no obvious clinical cause is found. However, several mechanisms have been hypothesized and supported by indirect evidence:

- Acute retinal ganglion cell death (neurotoxicity or apoptosis): One theory is that the stress of surgery (or the immediate postoperative environment) triggers a wave of retinal ganglion cell (RGC) death in an already compromised optic nerve. Unlike an infarction that happens all at once, this could be a cascading process of apoptosis in the remaining RGCs. A fascinating insight into this came from Mohammadzadeh et al. (2019), who used optical coherence tomography (OCT) to observe the macula in two eyes that had lost vision after glaucoma surgery [34]. They found progressive thinning of the macular ganglion cell layer over time, corresponding to the period of vision loss. This indicates that the wipe-out phenomenon in those cases was due to ongoing RGC degeneration (the central-most RGCs dying off), rather than an instantaneous event at the moment of surgery [34]. In other words, the surgery may have initiated a process that continued in the postoperative weeks—perhaps via loss of neurotrophic support, glutamate excitotoxicity, or some kind of optic nerve “shock” that caused cells already on the brink to perish [3,34]. The authors pointed out that progressive macular OCT changes can be a sign of snuff-out and provide evidence that wipe-out is essentially extreme glaucoma progression accelerated by surgical intervention [34]. This aligns with the clinical observation that sometimes the vision does not disappear immediately on post-op day 1 but declines over days to weeks and then stabilizes at no light perception [28,30].
- Ischemia–reperfusion injury: The act of drastically lowering the IOP in an eye that has adapted to a high IOP could cause a form of ischemia–reperfusion injury [8,30]. In advanced glaucoma, the optic nerve head may have chronically reduced perfusion due to a high IOP. When the IOP is suddenly normalized (or drops below normal in the case of hypotony), the sudden increase in perfusion pressure might paradoxically lead to damage—akin to reperfusion injury in other tissues [8,9]. Alternatively, if the IOP drops too low, transient optic nerve ischemia could occur due to insufficient intraocular pressure to support circulation (especially if blood pressure is low or autoregulation is impaired) [29]. This mechanism is hard to prove, but it is consistent with why extreme hypotony is dangerous and why gradual IOP reduction (say by staged procedures) might be safer in some cases. It has been likened to decompression sickness for the eye [18]. Decompression retinopathy, noted earlier, is one manifestation with retinal hemorrhages; wipe-out might be a variant where the damage primarily hits the optic nerve axons instead of manifesting hemorrhage [18,31].
- Optic nerve head circulation compromise: Related to the above, any factor that compromises the delicate blood supply to the optic nerve head can cause irreversible loss of the remaining function [8,31]. We mentioned retrobulbar anesthetic injections as a risk—these can cause direct trauma through the injection of anesthetic into the optic nerve sheath, leading to infarction of the optic nerve head or the occlusion of the central retinal artery [31]. Such an event would immediately wipe out vision. While this is not the classic mechanism in most wipe-out cases (most of which have uncomplicated anesthesia), it is a potential pathophysiology for some [18,31]. More broadly, patients with poor cardiovascular status might not tolerate fluctuations in ocular perfusion well [9,30]. No definitive studies show, for example, that low blood pressure intraoperatively causes wipe-out, but it is something surgeons keep in mind (maintaining adequate systemic blood pressure during sedation) [9].
- Choroidal effusion or maculopathy: If the surgery leads to a serous choroidal detachment or significant choroidal effusions (from hypotony), the macula can be distorted or the central retina compromised [10,29]. In most cases, hypotony maculopathy or choroidal detachments cause reversible vision loss (vision improves after the IOP normalizes and the retina reattaches). However, in an eye with advanced glaucoma, even a temporary insult to the macula might irreversibly silence the fragile remaining neurons [10,29]. Karadimas’s report of decompression retinopathy and other cases of massive choroidal effusions causing prolonged foveal dysfunction might overlap with wipe-out [18]. Essentially, structural changes from hypotony could conceivably push an already ischemic central retina over the edge [10,18,24].
- Pre-existing momentum of disease: One further consideration is that some eyes might have been destined to lose vision due to end-stage glaucoma progression, and surgery’s timing was coincidental [8,12]. The natural history of end-stage glaucoma can include sudden central loss (for example, from acute angle closure or late-stage optic disk changes). It is difficult to prove cause and effect. However, the temporal association with surgery in wipe-out cases (and the relative rarity of spontaneous “terminal events” in glaucoma without surgery) suggests that surgery is indeed a trigger in true wipe-out [10]. Mohammadzadeh’s OCT evidence strongly points to a causative role of surgery as the timing lined up and was bilateral in one of their patients only after surgeries [34].

In summary, the pathophysiology of wipe-out likely involves a combination of vascular and neurodegenerative processes. The common denominator is an optic nerve at the tipping point, which after surgery undergoes either an acute ischemic event or an accelerated apoptotic degeneration of its last functioning fibers [3,34]. This leads to the loss of central vision that is irreversible (distinguishing wipe-out from transient post-op vision loss, which can recover). Autopsy or histopathologic studies in wipe-out are virtually nonexistent (given how rare and idiosyncratic cases are), so our understanding comes from clinical deductions [10,18,24]. It is worth noting that wipe-out is not due to surgeon error or a recognizable intraoperative accident—by definition, the surgery is routine and intraocular structures appear fine [10]. This can be frustrating for surgeons and patients as they are left to infer invisible causes such as microcirculatory failure or cellular apoptosis [3,30].

### 3.4. Management and Prevention Strategies

Preventive measures are paramount since once wipe-out has occurred, there is no curative treatment to restore vision. Managing wipe-out is therefore about avoiding it in the first place and mitigating risk factors when operating on high-risk eyes. Based on the risk factors and mechanisms discussed, the following strategies have emerged:

- Careful patient selection and counseling: The surgeon must weigh the necessity of surgery in a patient with advanced glaucoma and very limited vision. If the eye in question is the patient’s only seeing eye (the other eye being blind), the decision is even more critical [10,24]. Modern evidence indicates that one should not categorically deny surgery due to fear of wipe-out—if glaucoma progression is threatening the remaining vision, surgery should be performed to control the IOP given the low incidence of wipe-out [9,18]. However, it is crucial to counsel the patient preoperatively about the small risk of catastrophic vision loss. Patients with advanced disease often understand that without surgery, they will also likely go blind; framing the discussion around relative risks is helpful [8]. In essence, “the risk of doing nothing is loss of vision from glaucoma, the risk of doing surgery includes a very small chance of immediate vision loss (wipe-out), but a much higher chance of preserving vision long-term.” Informed consent should include wipe-out as a potential complication in end-stage cases (even if one emphasizes its rarity). This conversation prepares the patient and manages expectations [8,10].
- Optimization of modifiable factors: Prior to surgery, any modifiable risk factors should be addressed [1,2]. For instance, if the patient has poorly controlled blood pressure, it should be optimized to ensure adequate ocular perfusion (avoiding extreme hypertension or hypotension) [30]. If the patient is on systemic beta-blockers or other meds that could lower blood pressure at night, the timing could be adjusted around the surgery to maintain perfusion. Some surgeons ensure the patient is well hydrated and has stable blood pressure on the day of surgery to reduce intraoperative ischemia risk. There is also a practice (anecdotally) of pre-treating with oral acetazolamide for a few days to gently lower the IOP before a big surgery so that the drop on the day of surgery is not so drastic—essentially to “soften the landing” for the optic nerve [18,29]. While not evidence-based by trials, this approach addresses the hypothetical reperfusion injury mechanism [1,2].
- Choice of anesthesia: To avoid potential optic nerve compromise from injections, many surgeons prefer sub-Tenon’s (subconjunctival) anesthesia or topical anesthesia for high-risk glaucoma cases [31]. Sub-Tenon’s (a blunt cannula infusion of anesthetic) anesthesia has a much lower risk of orbital hemorrhage or direct nerve trauma than retrobulbar injection [31]. Topical anesthesia with intravenous sedation avoids orbital injections entirely, though the patient must be cooperative. By eliminating retrobulbar needles, one removes a known, albeit rare, cause of catastrophic optic nerve injury [18]. If deeper anesthesia is needed (for instance, if a superior rectus bridle block is to be placed or the case is complex), many will still avoid inserting the needle too deeply [31]. In short, the anesthetic technique should be tailored to minimize risk in these eyes [18,31].
- Surgical technique to minimize hypotony: In an eye at risk for wipe-out, the surgeon can employ measures to prevent extreme IOP lows [10]. For trabeculectomy, this might include using a smaller scleral flap or leaving more sutures tied, with a plan to gradually lower the IOP [6]. The use of releasable or adjustable sutures is invaluable—one can leave the flap fairly secure on day 1 (to avoid very low pressures) and then incrementally loosen sutures in the clinic to reach the target IOP over a week or two [32]. Anti-fibrotic use (mitomycin-C) should be balanced: enough should be used to achieve long-term IOP control but not so much that the flap undergoes filtration too fast [6,32]. In very high-risk eyes, some surgeons even stage the procedure: for example, they first perform cataract extraction, which often lowers the IOP a bit in glaucoma patients, and then trabeculectomy is conducted a few weeks later once the eye has healed [21]. The idea is to reduce the IOP in multiple steps rather than all at once. Another approach described is performing a limited pars plana vitrectomy at the time of trabeculectomy to absorb some fluid and avoid sudden shallowing of the anterior chamber (though this is not common practice). In tube shunt surgery, one can choose a valved implant (e.g., Ahmed valve) that prevents hypotony [35], or if using a non-valved implant (e.g., Baerveldt), the tube is ligated for the first 4–6 weeks so that the IOP is gradually reduced as the ligature dissolves [26]. These techniques ensure that the IOP does not plummet to near-zero on day 1, thus protecting against hypotony-related mechanisms [10,29]. The importance of this is highlighted by Costa’s finding of wipe-out in eyes with a day-1 IOP of 2 mmHg—something we now have the tools to avoid [10].
- Consideration of MIGS or alternate surgery: For some advanced patients, especially if the IOP is not extremely high, a surgeon might consider an MIGS or less invasive procedure in lieu of a trabeculectomy, specifically to mitigate wipe-out risk [13,14]. For example, some case series have tried GATT or canaloplasty in advanced glaucoma as a primary surgical option, precisely because these ab interno approaches do not typically cause hypotony [20,25]. A 24-month GATT study in advanced glaucoma showed good IOP control in ~77% of eyes and only one wipe-out case (2.3%), suggesting that it can be a viable alternative to trabeculectomy for certain patients [25]. Another example is deep sclerectomy or viscocanalostomy in advanced glaucoma—by avoiding entering the eye, these have a smoother postoperative course (Leleu et al. reported no sudden vision loss in severe glaucoma with non-penetrating surgery) [19]. Cyclophoto-coagulation (laser treatment to ciliary body) is also an option to lower the IOP without incisional surgery; micropulse transscleral laser in particular has a milder effect and could be considered for frail eyes [36]. However, one must balance efficacy—MIGS or laser might not lower the IOP enough in very advanced cases, risking ongoing glaucoma damage [15,37]. Increasingly, glaucoma specialists individualize the plan (as advocated by Mégevand and Bron’s personalized surgery approach): an ab interno MIGS might be attempted first for moderate control, and a filtering surgery can be reserved as a second step if needed [15,33]. The good news is that MIGS has expanded our surgical toolkit so we can attempt lower-risk interventions first in many cases [16,17].
- Intraoperative and postoperative vigilance: During surgery, the surgeon will be attentive to avoid long hypotensive episodes or excessive manipulation. For instance, preventing a long period of eyeball compression is relevant—older anesthesia involved a Honan balloon to soften the eye, but one wouldn’t use that in an end-stage eye for long [31]. Postoperatively, close monitoring is critical. The surgeon should see the patient early (often on day 1 and day 3 rather than waiting a week) to check the IOP and the eye’s status. If the IOP is very low and the patient has choroidal effusion or complains of vision decline, one should intervene promptly (e.g., place a suture in the trabeculectomy flap, inject viscoelastic to raise the IOP, etc.) [10,29]. Treating any complications aggressively—such as through choroidal detachments, bleb leaks, or hypotony—may prevent a transient problem from becoming permanent [2,18,38]. There is some evidence that even if central vision is lost immediately, visual recovery can occur if the cause is transient. Francis et al. (2011) observed that transient vision loss after trabeculectomy is common and may take up to 1–2 years for recovery, whereas truly permanent vision loss is less common [38]. This means that some patients who seem to have a “wipe-out” initially might actually improve if the issue was hypotony maculopathy or cataract [5,6]. Therefore, distinguishing true wipe-out (irreversible) from temporary loss (reversible) is part of management [29,38]. In practice, if a patient’s vision drops after surgery, one would conduct a thorough exam to rule out the possible causes, determining whether the cornea is cloudy, whether the lens is swollen, whether there is vitreous hemorrhage, whether the retina is attached, the nerve’s appearance, etc. If nothing is obvious and the IOP is adequate, observation is warranted with supportive measures like corticosteroids (to reduce any inflammation) and perhaps neuroprotective vitamins [3,4]. Unfortunately, if after a few months there is no improvement and no pathology, one has to conclude that it was wipe-out [10].
- Rehabilitation and support: In the unfortunate event that a wipe-out does occur, management should focus on low vision rehabilitation and patient support. The patient will need counseling to cope with sudden blindness. Orientation and mobility training, low-vision aids (if any vision remains in the periphery or light perception), and connecting with vision loss resources are crucial [29,39]. While this is outside the scope of surgical management, it is an important aspect of caring for the patient post-complication. Psychologically, wipe-out can be devastating (the patient often questions why they had surgery). It is incumbent on the clinician to provide empathy, a clear explanation (while acknowledging the limits of our understanding), and continued care, including perhaps second opinions to confirm nothing treatable was missed [8,24].

It should be emphasized that the high success rates of modern glaucoma surgery far, far outweigh the minute risk of wipe-out. By employing the above strategies, surgeons have drastically reduced the incidence of wipe-out. Indeed, many glaucoma surgeons with decades of experience have never personally seen a wipe-out case, or perhaps only one [5,38]. This rarity, however, does not make it a myth—rather, it is a testament to careful surgical practice which has kept it so rare. The community still shares case reports and cautionary tales (like Ozmen’s MIGS case) to ensure that we remain vigilant [25].

## 4. Discussion

From the evidence gathered, wipe-out in glaucoma surgery is an exceedingly rare phenomenon—so rare that its very existence was debated, yet so impactful that it cannot be ignored. The question “should we fear wipe-out?” must be answered with nuance. On one hand, the incidence is so low with modern techniques that routine fear might be disproportionate [5,8,18]. On the other hand, for those few patients who do experience it, the consequences are catastrophic, and thus, consideration of this complication is warranted, especially when operating on high-risk eyes [9,10].

Regarding its relevance in the modern era, the collective data suggest that wipe-out remains a possible complication but is far less common than historically perceived [11,18]. In the era of trabeculectomy augmented with antifibrotics and the introduction of MIGS, the environment in which wipe-out was first described has changed. The early fear of wipe-out largely stemmed from small case series and theoretical concerns in the 1980s [8,12]. Surgeons back then lacked the finesse in controlling early postoperative IOP that we have now. As a result, there was perhaps an overestimation of the wipe-out risk, leading to an entrenched cautious attitude [10,18,38].

Contemporary studies, particularly prospective ones, have essentially debunked the notion that wipe-out occurs with any appreciable frequency when surgery is performed properly. Topouzis et al.’s finding of zero cases in end-stage trabeculectomy patients was a pivotal reassurance [9]. Likewise, the TAGS trial outcome—showing that performing surgery in advanced cases did not worsen patient-reported outcomes and indeed controlled glaucoma better—directly challenges the idea that one should avoid surgery for fear of wipe-out [7]. In the TAGS and similar studies, if wipe-out were common, the surgical arm would have shown many patients with profound vision loss, which it did not. In fact, in the TAGS, the surgery arm had better visual field preservation (implying that timely surgery prevented glaucoma progression without causing wipe-out in those patients) [7].

Therefore, one could argue that the risk of wipe-out has been overstated in the past [8,11]. Some authors have called it a “blast from the past,” implying that it is a complication largely of historical interest and not something that should keep surgeons awake at night today [8]. Dr. Murray Moster, in his 2005 commentary, effectively said as much—that wipe-out might be more myth than reality in the context of modern practice [8].

The advent of MIGS has indeed transformed glaucoma surgery, but mostly for earlier stages of disease [13,16]. MIGS has allowed intervention at a point when patients still have moderate vision, thereby potentially reducing the number of patients who progress to end-stage disease without surgery [17,20]. For example, a patient who might have gone on maximal drops until very advanced might now receive an iStent or Hydrus implant when they have moderate glaucoma, achieving some IOP control earlier. This could mean fewer patients reaching that “point of no return” state where a high-stakes trabeculectomy is the only option. In that sense, MIGS indirectly lowers the incidence of wipe-out by lowering the frequency of surgery in end-stage eyes [14].

For patients who reach end-stage, MIGS offers alternative surgical pathways that might carry less wipe-out risk. The safety profile of MIGS is undeniably superior to traditional surgery in terms of serious complications [14,16]. The absence of bleb and hypotony in many MIGSs virtually eliminates classic wipe-out triggers [16,20]. We have now seen that even MIGS is not absolutely foolproof (the GATT case), but that single case underscores how rare it is—considering that tens of thousands of MIGSs have been performed worldwide by now, a single published wipe-out is actually evidence of how low the risk is [25]. Additionally, some MIGSs like the XEN gel stent or PreserFlo microshunt create blebs and can cause hypotony, yet even with those, reports of wipe-out are extremely scarce [33,37]. One recent study of the PreserFlo MicroShunt in refractory glaucoma noted one case (out of ~12 patients) of “wipe-out syndrome due to clinical hypotony”, which again highlights hypotony as a common thread [37]. But broadly, MIGS has made glaucoma surgery safer and likely virtually eliminated wipe-out in mild to moderate glaucoma cases [14,15].

It is also notable that MIGS has spurred a cultural shift in glaucoma surgery—surgeons are now more accustomed to intervening surgically earlier, and this perhaps lessens the psychological fear of surgery in general. In the past, surgery was seen as a last resort partly because of catastrophic risks like wipe-out or infection [8,12]. With MIGS making surgery seem “gentler,” surgeons might be less inclined to postpone surgery until the end stage. Thus, the scenario that produces wipe-out (surgery on the only eye with remaining vision) may be encountered less frequently if patients are already being managed surgically before reaching that point. This is speculative but plausible in a real-world sense [14,33].

Is the risk overstated or still relevant? For the majority of glaucoma surgeries being performed today (which are performed for early to moderate disease, often combined with cataract), wipe-out is practically a non-issue [16,20]. It is not something discussed when using a combined phaco-iStent on a patient with mild glaucoma—the risk there is effectively zero [16]. Where it remains relevant is in the subset of very advanced glaucoma cases, which are still encountered [9,10]. In those cases, every surgeon still pauses and meticulously plans because of the risk of wipe-out (among other concerns). The collective experience of glaucoma specialists affirms that wipe-out, while extremely rare, can happen in any era [8,9]. All it takes is a perfect storm of risk factors. Therefore, the community still “fears” wipe-out in the sense that it is a known specter when operating on an eye with only a thread of vision left [8,9].

However, given the evidence, this fear should be kept in proper perspective. It should not lead to therapeutic paralysis or to denying beneficial surgery [14,18]. The high success rates and vision-preserving outcomes of glaucoma surgery in advanced patients far outweigh the minuscule wipe-out risk. In concrete terms, if an advanced glaucoma eye has a high IOP that will surely cause blindness in a year, the perhaps 1% (or less) chance of wipe-out with surgery is worth taking to potentially save the remaining vision [18]. As the results show, modern series indicate that the percentage of incidence might even be closer to 0% with careful management [9,10]. So, the fear of wipe-out has likely been too strong historically, and current evidence-based practice encourages us to proceed with necessary surgeries while using strategies to mitigate risk [10,18].

There are differences across surgical techniques. Comparing wipe-out risk across various glaucoma surgeries, trabeculectomy in an end-stage eye historically had the highest association simply because it was the most common surgery performed in those eyes. With refined techniques (tight flap and gradual titration), trabeculectomy outcomes now show essentially no significant wipe-out incidence (e.g., none in Topouzis’s 21 eyes) [9]. Tube shunt surgery was sometimes chosen by surgeons in lieu of trabeculectomy for very advanced eyes under the belief that it might reduce wipe-out risk (especially valved tubes that avoid hypotony) [26,34]. While no head-to-head data exist specifically on wipe-out rates, in practice, tubes have not been noted to cause wipe-out often, and they offer the advantage of a more controlled early IOP [7,26]. The TAGS did not report any wipe-out cases in tube patients, so one could infer that the risk is similarly very low [7]. Non-penetrating surgeries and MIGSs likely carry the lowest risk of all as they inherently avoid the mechanisms thought to cause wipe-out (no sudden pressure drop, no bleb-related hypotony, and minimal inflammation) [14,19,36]. Indeed, it is telling that wipe-out is so rare now that when one occurs after an MIGS, it is publishable as a case report—reinforcing how exceptional it is [25].

When comparing patient profiles, it can be seen that patients who suffer wipe-out tend to be those with far advanced disease [10,24]. An interesting question is whether normal-tension glaucoma (NTG) patients, who often have vasospasm issues, might be more prone to wipe-out. There is scant direct evidence, but one might speculate that an NTG patient’s optic nerve, already susceptible to low perfusion, could be at risk if the IOP is drastically altered [1,34]. Conversely, an angle-closure glaucoma patient who is very advanced might have other issues like chronic ischemia [2,29]. The case reports we reported (Costa’s wipe-outs, Mohammadzadeh’s cases, and Ozmen’s case) do not particularly point to one subtype—they include pseudoexfoliation, primary OAG, etc. [10,25,34]. So, it appears to be more about stage than subtype [10,24].

High success rates vs. rare risk: Ultimately, we must emphasize that modern glaucoma surgeries, including trabeculectomy and tube shunts, have very high success rates in controlling the IOP and preventing further vision loss [7,10,18]. The risk of severe complications is low. For instance, trabeculectomy carries perhaps a ~5% risk of infection or serious complications over a lifetime and an even lower risk of wipe-out [10,18,38]. MIGSs have even lower serious complication rates (near zero for phenomena like endophthalmitis or wipe-out) [15,16,37]. The benefit–risk calculus strongly favors surgery in those who need it. It would be a disservice to patients to hold back a needed operation due to an outsized fear of wipe-out. The literature and expert consensus now support being proactive with surgery when indicated, using MIGS when suitable and not hesitating to perform a trabeculectomy in an advanced eye that requires it [15,37,38]. The American Glaucoma Society’s position paper on MIGS (2020) makes it clear that MIGS can be an important tool but should not completely replace trabeculectomy/tubes in advanced disease—rather, one should choose the right procedure for the patient [37]. If MIGS will not achieve a low enough IOP in an end-stage eye, trabeculectomy is still the gold standard, and evidence shows that it can be performed safely [9,10,38].

Regarding wipe-out vs. progression, it is interesting to note that patients themselves sometimes conflate wipe-out with normal glaucoma progression [10,24]. A patient who loses vision slowly from glaucoma might wonder whether a prior surgery caused it (when in fact it was the disease) [10]. Conversely, if a patient loses vision after surgery, distinguishing wipe-out from aggressive progression can be challenging [10,24]. We rely on timing (sudden vs. gradual) and exam findings. In any event, one must also consider that performing surgery too late in the disease course is a problem—if an eye is one week away from natural wipe-out due to glaucoma, operating at that point might be blamed for the loss when in reality, it was inevitable [9,10]. This is another reason to intervene earlier rather than later when possible [9,10].

Regarding the perspective of experienced surgeons, anecdotally, many seasoned glaucoma surgeons will say that wipe-out is something they always keep in the back of their minds but have rarely if ever seen it [8,18]. This aligns with the low incidence reported in the literature [9,10]. It also suggests that our preventive strategies have been effective [5,10,18]. It is telling that in the Costa 1993 series, only 4 out of 508 eyes had wipe-out—even at that time, it was uncommon [10]. Now, with even better techniques, it is arguably rarer. Thus, while we respect the possibility of wipe-out, we do not fear it to the point of allowing it to change our overall management approach. Instead, we approach high-risk cases with appropriate caution and technique adjustments, but we proceed because the vast majority of such surgeries greatly benefit the patient [5,10,18].

To enhance surgical results and reduce the likelihood of severe vision loss, we have underscored the need for the early detection of high-risk patients and prompt intervention. Patients exhibiting extensive glaucomatous damage, especially those with split fixation or limited visual reserve, necessitate a comprehensive preoperative dialog that weighs the imperative for intraocular pressure reduction against the safest and most suitable surgical approach. Evidence increasingly supports the execution of minimally invasive glaucoma surgery (MIGS) or less invasive operations prior to the advancement of disease to an end-stage level, thereby conserving visual function and circumventing high-risk situations. A tailored, patient-focused methodology based on visual field assessment, optic nerve capacity, and systemic risk determinants enables clinicians to recommend and execute filtering surgery prior to the onset of irreversible visual impairment. This strategy not only reduces wipe-out danger but also conforms to modern ideas of proactive and preventive glaucoma management.

## 5. Conclusions

Wipe-out is rare in modern times, especially when proper surgical techniques are used. Previous studies estimated wipe-out rates of 8–13% in high-risk cohorts, but 21st-century prospective data and comprehensive reviews suggest a significantly lower rate of 0–1% or almost zero in several series. Due to their delicate vision, advanced glaucoma eyes are at risk, although surgery and careful management can maintain vision. We detected substantial preoperative visual field loss in the central field, older age, and perioperative optic nerve hypoperfusion or shock (such as extreme hypotony) as wipe-out risk factors. Surgeons can prevent rapid IOP dips and ensure optic nerve perfusion by understanding these factors. Wipe-out is likely caused by acute vascular insufficiency and rapid ganglion cell death, as evidenced by increasing macular thinning on OCT. This condition may be the abrupt end of glaucoma caused by surgical stress in an end-stage eye.

Importantly, minimally invasive glaucoma surgeries (MIGSs) have improved surgical safety. MIGSs have a high success rate in decreasing the IOP and a good safety profile, with practically no wipe-out in clinical trials. MIGS and other contemporary techniques have reduced glaucoma-related surgical complications and allowed surgeons to intervene earlier, potentially lowering the need to operate on the last eye with vision. Canal-based MIGS or non-penetrating operations can reduce danger in advanced instances, but classic filtering surgery is best for very low target pressures. Current evidence shows that with careful technique (adjustable sutures, staged pressure reduction, etc.), wipe-out is extremely rare and likely outweighed by the risk of not treating high pressure.

Wipe-out is a significant consequence but should not be feared enough to delay glaucoma surgery. Data and clinical experience show that wipe-out is unlikely, but glaucoma surgical success (preventing additional vision loss) is high. Modern MIGS and classic glaucoma operations have high success rates in managing IOP and preserving vision, showing improved safety profiles. In patients who need surgery, limiting glaucomatous damage outweighs the uncommon danger of wipe-out. Modern glaucoma surgery’s great success rates outweigh its extremely low wipe-out risk. Surgeons should be careful in high-risk situations and reduce risk, but they can reassure patients that wipe-out is rare. We can minimize this consequence by staying up to date on surgical advancements and evidence-based methods. In conclusion, wipe-out is real but unusual, and with contemporary MIGS and better filtering surgeries, it has become more of a historical footnote than a typical fate. To save vision, glaucoma should be treated properly; it is treated in most cases without incidents because of developments in surgical technology and technique.

## Figures and Tables

**Table 1:** Risk factors related to wipe-out.

Risk Factor	Description/Mechanism
**Advanced visual field loss (split fixation)**	Glaucoma encroaching on the central field (e.g., split fixation or only a small central island) leaves the optic nerve with minimal reserve; any further insult (e.g., sudden IOP drop) can abolish the remaining vision [5,29].
**Older age**	Older patients are more likely to experience wipe-out; aging may reduce optic nerve autoregulation and perfusion resilience, making nerves more susceptible to IOP fluctuations [5].
**Early postoperative hypotony**	Immediate postoperative hypotony (very low IOP) can severely compromise ocular perfusion and induce structural changes (e.g., choroidal effusions and retinal folds) that damage central vision [5,6].
**Perioperative ocular ischemia**	Ischemic insults during or after surgery (e.g., optic nerve or retinal artery compromise from anesthesia or systemic vascular disease) can further reduce perfusion to an already compromised optic nerve, precipitating wipe-out [1,7].
**Optic nerve vulnerability**	Severely damaged optic nerves (thin neuroretinal rim and extreme cupping) are intrinsically fragile; even minor surgical stress can cause an abrupt loss of the remaining ganglion cells (especially in high myopia) [25].
**Type of surgery/surgical technique**	Procedures causing abrupt IOP reduction (e.g., uncontrolled full-thickness trabeculectomy) historically had a higher wipe-out risk; modern guarded or non-penetrating techniques (adjustable-suture trabeculectomy, deep sclerectomy, and MIGS) allow gradual decompression and have much lower risk [3,4,17].

## Data Availability

No new data were created or analyzed in this study. Data sharing is not applicable to this article.
